# Prevalence of Lungworm Infections in Hedgehogs (*Erinaceus roumanicus*) in Greece and a Novel Therapeutic Approach for Treatment

**DOI:** 10.3390/pathogens15010011

**Published:** 2025-12-21

**Authors:** Grigorios Markakis, Isaia Symeonidou, Anastasia Komnenou, Frederic Beugnet, Maria Ganoti, Elias Papadopoulos

**Affiliations:** 1 ANIMA—The Hellenic Wildlife Care Association, 10442 Athens, Greece; grigmark@vet.auth.gr (G.M.); maria.ganoti@gmail.com (M.G.); 2 School of Veterinary Medicine, Faculty of Health Sciences, Aristotle University, 54124 Thessaloniki, Greece; isaia@vet.auth.gr (I.S.); natakomn@vet.auth.gr (A.K.); 3 Boehringer Ingelheim Animal Health, 69007 Lyon, France; frederic.beugnet@boehringer-ingelheim.com

**Keywords:** *Capillaria*, *Crenosoma*, eprinomectin, esafoxolaner, Greece, hedgehogs, praziquantel

## Abstract

The most common causes of respiratory diseases in wild hedgehogs are the lungworms *Crenosoma striatum* and *Capillaria aerophila*, which can lead to life-threatening pneumonia. The aim of the current study was (A) to assess the prevalence of common lungworm infections in northern white-breasted hedgehogs (*Erinaceus roumanicus*) in Greece and (B) to identify an efficient and easy to administer treatment option. (A) Fifty-six hedgehogs were admitted to a Greek wildlife rehabilitation center and included in the present study. Fecal tests were performed using the flotation method (ZnSO_4_ 33.2%) combined with the Baermann technique. In total, 49 hedgehogs excreted *Crenosoma* spp. larvae (87.5%), and 27 of them were also infected with *Capillaria* spp. (48.2%). One of them died, and the necropsy and lung histopathology confirmed the diagnosis of crenosomosis. (B) Fourteen animals with mixed infections were treated using NexGard^®^ Combo (esafoxolaner, eprinomectin, praziquantel) administered orally at a dose rate of 0.2 mL/kg body weight, once. On days 10 and 14 post-treatment, no parasitic elements were detected in the feces of the infected animals. All the animals had gained weight by day 14, and their biochemical parameters were normal. It was concluded that this combination given orally was safe and successful against hedgehog respiratory nematodes.

## 1. Introduction

The European hedgehog (*Erinaceus europaeus*) is one of the most common European wild mammalian species, and its close relative, the northern white-breasted hedgehog (*Erinaceus roumanicus*), is widespread in Greece. Human-related causes and infectious conditions have resulted in their populations tending to decline [1]. More specifically, 59% of hedgehog deaths are due to various diseases [2], including infections of the respiratory tract.

Lungworm infections are the most common cause of respiratory disease in European hedgehogs [3]. The nematodes *Crenosoma striatum* and *Capillaria aerophila* are the hedgehog’s most common pulmonary parasites [1,4]. Lungworms are transmitted both through gastropods, which hedgehogs feed on, and also directly [5,6]. Lungworm infection can be subclinical or cause lethargy, inappetence, weight loss, respiratory clinical signs, and severe pneumonia, which is usually complicated by bacterial infections and if left untreated, it can be life-threatening [3,7].

Moreover, it is noteworthy that, although the hedgehog is the only known final host for *C. striatum*, *C. aerophila* also parasitizes dogs and cats, as well as other wild animal species [1], and is potentially zoonotic [8].

In view of the aforementioned data, the wildlife rehabilitation centers that take care of hedgehogs should ensure the precise diagnosis and treatment of these lungworm infections. As of now, the therapeutic solutions remain limited, as they are empirical and not based on scientific evidence [1]. Clinicians, especially those who work with wildlife, would rather use easily administered and broad-spectrum antiparasitics. NexGard^®^ Combo (Boehringer Ingelheim, Germany) is a commercial preparation that acts against a broad spectrum of endo- and ectoparasites, by combining three active ingredients: esafoxolaner, eprinomectin, and praziquantel.

In the present study, cases of lungworm infections in northern white-breasted hedgehogs (*E. roumanicus*) in Greece are described, and a novel therapeutic approach is presented.

## 2. Materials and Methods

### 2.1. Study Design

In total, 56 hedgehogs that were found wandering in urban areas during June to August 2025 were admitted to ANIMA—The Hellenic Wildlife Care Association (Athens), a licensed wildlife hospital, which is officially authorized to perform veterinary procedures (license number ΥPEN/DDD/54344/1692). During the first part of the study, all animals were sampled and examined individually for the presence of lungworms. One hedgehog, which presented with nasal discharge, died, and necropsy and lung histopathology were performed. Adult parasites collected during the necropsy were identified using the morphological keys described by [5].

Thereafter, 14 infected hedgehogs were randomly chosen from the parasitized ones and were included in the second part of the study to assess the efficacy of treatment with esafoxolaner, eprinomectin, and praziquantel (NexGard^®^ Combo Boehringer Ingelheim, Ingelheim, Germany). NexGard^®^ Combo was administered orally on day 0. On days 7, 10, and 14 post-treatment, fecal specimens were collected and examined individually for the assessment of the treatment’s efficacy.

Furthermore, on days 0 and 15, 0.5 mL of blood was collected from the medial saphenous vein of each hedgehog under anesthesia (mask induction of 5% isoflurane in an oxygen flow rate of 2 L/min and then maintenance in 2% isoflurane). The blood was sent to a specialized laboratory (LABOKLIN, Bad Kissingen, Germany) for measuring biochemical parameters to assess the renal and hepatic function of the hedgehogs pre- and post-treatment. More specifically, blood urea nitrogen (BUN), creatinine (Crea), alanine transaminase (ALT), and glutamate dehydrogenase (GLDH) were measured. The body weight of each animal was also recorded on days 0 and 15.

During the study, all the hedgehogs were hospitalized individually in pet carriers, under the same and controlled environmental conditions (25 °C, humidity 40%), and were fed with a mixture of cat food and live mealworms.

### 2.2. Fecal Sample Collection and Coprological Methods Employed

The fecal samples were collected fresh from each hedgehog, directly from the floor of the pet carrier where the animals were hospitalized. All specimens were put in plastic containers individually, tagged, and stored at 4 °C until they were examined (within 2 days) at the Laboratory of Parasitology and Parasitic Diseases of the School of Veterinary Medicine of Aristotle University (Thessaloniki). Each sample was tested via the flotation method using ZnSO_4_ (33.2%) in combination with the Baermann method, as described by [9], to assess the presence of parasitic elements.

More specifically, the fecal samples (approximately 1–2 g each) were mixed with 10–12 mL of water in test tubes individually and stirred with a wooden stick to create uniform suspensions, which were then strained through a stainless-steel mesh into separate 15 mL test tubes to remove large debris, which was discarded. Then, the test tubes were filled to the top with water, and then the samples were centrifuged (1500 rpm for 3 min) to further separate the heavier particles. Afterwards, the supernatant was discarded, and the tubes were filled halfway with a solution of 33.2% zinc sulfate, and the sediments were stirred with wooden applicator sticks before the tubes were filled almost to the top with the same solution. The tubes were placed in the centrifuge and, using a dropper, the flotation solution was added until a meniscus was formed on the top of the tubes. An 18 × 18 mm cover slip was placed οn each meniscus and the samples were recentrifuged (1500 rpm for 3 min). Finally, the cover slips were immediately removed by lifting vertically, placed onto microscopic slides, and tested microscopically (at 100× and 400× magnifications) in a meander pattern for the detection of the parasites. This is a scanning pattern characterized by alternating horizontal sweeps with small vertical shifts, ensuring the entire surface is examined without overlap or gaps.

For the Baermann technique, warm water (approximately 25 °C) was placed in a glass funnel equipped with a stopcock on a rubber hose at its lower end. Approximately 5 g of feces were wrapped in two layers of gauze and then submerged in the water within the funnel. It was left there for approximately 8 h for the L1 larvae to migrate, and afterwards, 10 mL of fluid was withdrawn from the bottom of the funnel and centrifuged at 1500 rpm for 5 min. Finally, using a pipette, 3 drops were extracted from the bottom of the centrifuge tubes and transferred onto a microscope slide. Then, a coverslip was applied, and they were examined microscopically (at 100× and 400× magnifications) for the isolation of lungworm larvae.

The microscopic identification of the parasitic elements was based on the morphological criteria of the lungworm stage 1 larvae infecting hedgehogs, as described by Bexton [10].

### 2.3. Treatment Administration, Acceptability of Treatment, Safety Evaluation, and Statistical Analysis

On day 0, 14 randomly selected hedgehogs infected with both *Crenosoma* and *Capillaria* were weighed and treated using a combination of esafoxolaner, eprinomectin, and praziquantel (NexGard^®^ Combo, Boehringer Ingelheim, Ingelheim, Germany) per os at a dose rate of 0.2 mL/kg BW, once. At this dosage, NexGard^®^ Combo delivers 1.4 mg/kg esafoxolaner, 0.5 mg/kg eprinomectin, and 5 mg/kg praziquantel.

All hedgehogs that were included in the treatment protocol were subjected to a clinical examination (posture, behavior, gait, body condition score, respiration pattern, rectal temperature, heart and respiratory rate, color of mucous membranes, capillary refill time, and hydration status) on days −3, 0, 7, 10, and 14. Moreover, the rehabilitators closely observed the animals for approximately 3 h post-treatment and then twice daily, until the end of the study, in order to document any adverse events. Finally, as described in Section “2.1. Study Design”, to further assess the safety of the treatment, certain biochemical parameters were measured in the blood before the beginning and after the end of the treatment.

Statistical analysis was performed using a *t*-test comparing pairs (D0 and D15). The level of significance was set at *p* < 0.05.

## 3. Results

**Figure 1:**
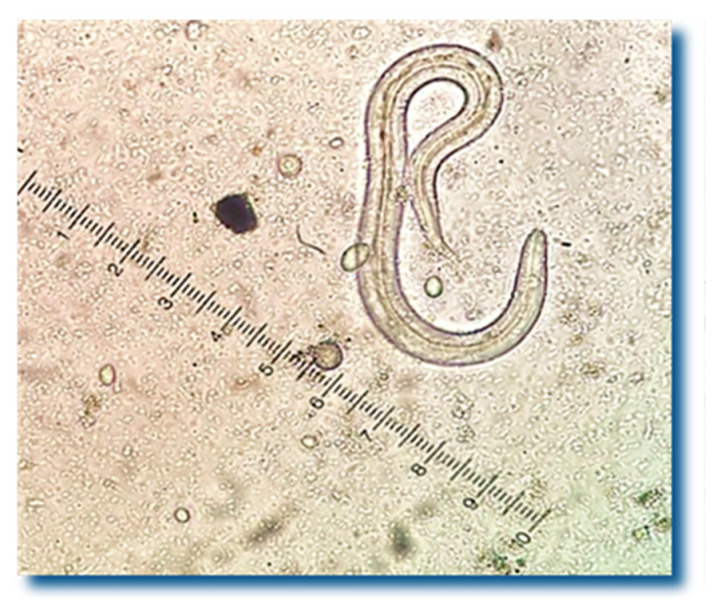
*Crenosoma* spp. larvae were detected in 87.5% of the hedgehogs’ fecal samples.

**Figure 2:**
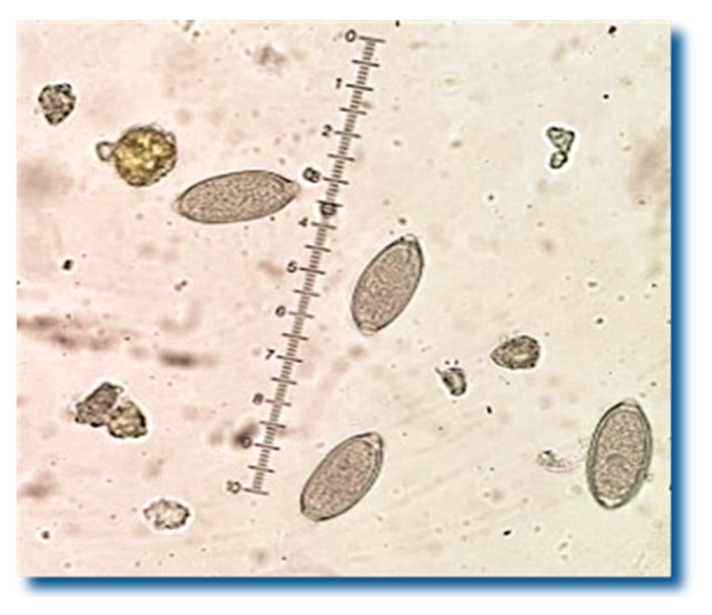
*Capillaria* spp. eggs were detected in 48.2% of the hedgehogs’ fecal samples.

*Crenosoma* spp. larvae were detected in the feces of 49 hedgehogs out of 56 (87.5%) (Figure 1), and 27 of them (48.2%) also excreted *Capillaria* spp. eggs simultaneously (Figure 2).

**Figure 3:**
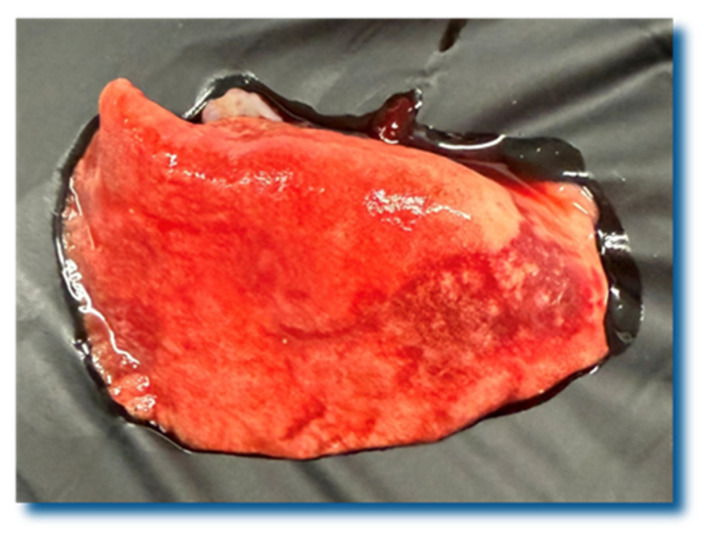
Gross pathology of the lung of a northern white-breasted hedgehog (*E. roumanicus*) with crenosomosis. Note the moderate enlargement with areas of consolidation and multifocal pyogranulomatous foci in the cranioventral lobes.

**Figure 4:**
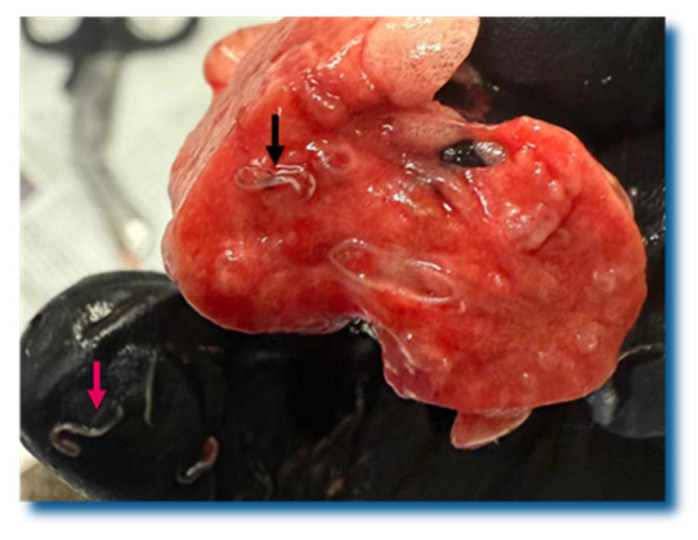
Cross-section of the lung parenchyma of a northern white-breasted hedgehog (*E. roumanicus*) with crenosomosis. Multiple sectioned nematodes (arrows) are present in the bronchiolar lumens.

The necropsy of the hedgehog that died shortly after its admission revealed lung lesions consistent with bronchopneumonia. The lungs were moderately enlarged and the cranioventral lobes showed areas of consolidation and multifocal pyogranulomatous foci (Figure 3). The cross-section of its parenchyma showed nematodes that resembled *Crenosoma* spp. (Figure 4). The histological examination (hematoxylin and eosin staining) confirmed the diagnosis of mixed cellular, extensive, and moderate bronchopneumonia, with intralesional *C. striatum* nematodes. The bronchiolar epithelium was mildly hyperplastic, and infiltration of mixed leucocytes and rare eosinophils was observed. There were foci of atelectasis and also some of mild alveolar hyperinflation. The *Crenosoma* in the bronchiolar lumens were approximately 300 µm in diameter. They had eosinophilic cuticles, polymyarian coelomyarian musculature, a pseudocoelom, and a digestive tract lined by few multinucleate cells with a short brush border. Occasionally, brown pigment was present in their digestive epithelium. Some of the *Crenosoma* showed a uterus distended by ova and larvae.

After treatment of 14 hedgehogs on day 0, no parasitic elements were detected in the feces of the 13 out of the 14 treated hedgehogs on days 7, 10 and 14. Only one hedgehog still had *Crenosoma* spp. larvae on day 7 post-treatment but became negative on days 10 and 14.

The curative efficacy after a single oral administration of the formulation containing eprinomectin was 100% against both *Crenosoma* spp. and *Capillaria* spp.

All the clinical examinations which were carried out during the course of the study were unremarkable, and no adverse effects related to the treatment were observed. Regarding the tested biochemical parameters (BUN, Crea, ALT, GLDH), all were categorized as normal in all hedgehogs, both before and after the treatment, according to the blood reference intervals for *E. europaeus* [11].

All the treated hedgehogs gained weight during the 15 days of the study, with a significant (*p* < 0.05) mean gain weight of 25% (Table 1).

## 4. Discussion

In the present study, lungworm infections by *Crenosoma* spp. and *Capillaria* spp. in northern white-breasted hedgehogs in Greece were described, and their occurrence was evaluated. The detected proportion of *Crenosoma* infection was notably high (87.5%). Remarkably, several studies conducted in Europe have detected lower prevalences. In a previous survey that investigated the endoparasites of wild mammals in Greece, the prevalence *C. striatum* infection was 47.4% in European hedgehogs [12]. Furthermore, a recent study detected a 41% occurrence of *C. striatum* in hedgehogs presenting to a British wildlife rehabilitation center [3]. An older study detected a 56.6% prevalence of infection by *Crenosoma* spp. in the UK [6]. Noteworthily, *C. striatum* was the most frequent endoparasite isolated in hedgehogs in Italy and Great Britain, and the infection proportions were 45% and 51%, respectively [13,14]. In a study conducted in Portugal, *C. striatum* was observed in the lungs of 45.5% of the hedgehogs studied [5]. Finally, the prevalence of *C. striatum* in European hedgehogs in Denmark, determined by detecting the larvae in intestine during necropsy, was lower (32.1%) [4]. This discrepancy could be attributed to the intermittent fecal shedding of the first-stage larvae, in addition to the examination of only a single initial fecal sample [3].

As far as *Capillaria* spp. is concerned, in the current study, approximately half of the hedgehogs excreted *Capillaria* spp. eggs in their feces (48.2%). This is in accordance with a survey conducted in Denmark, where eggs of *Capillaria* spp. were detected in the intestinal contents of 51.6% of the hedgehogs studied [4]. Nevertheless, some surveys have recorded lower rates of infection (36.8–42.5%). Specifically, 36.8% of the European hedgehogs examined in Greece in 2017 were infected with *Capillaria* spp. [12]. In the same context, 39.6% of British hedgehogs were found to be infected with *Capillaria* spp. [6]. Remarkably, in 2010, 62% of the hedgehogs studied in Great Britain were found to excrete *Capillaria* spp. eggs in their feces [13]. Noteworthily, a recent study demonstrated a significantly higher prevalence (81%) [3].

Regarding mixed infections, in the present study, almost half of the tested hedgehogs had a mixed infection with *Crenosoma* spp. and *Capillaria* spp. (48.2%), which is consistent with the results of a study conducted in the UK (53.3%) [6]. Notably, in a recent survey conducted in the same country, the occurrence of mixed infections was lower, as 37% of the hedgehogs were infected by both *Crenosoma* spp. and *Capillaria* spp. [3].

*Crenosoma striatum* and *C. aerophila* infect the trachea and bronchi and can cause severe clinical signs in hedgehogs, including respiratory signs, anemia, and even death in cases of high parasite burdens [15]. Furthermore, there is a potential threat to humans [8,16], as people may be infected by *C. aerophila* by consuming the eggs of the lungworm located on contaminated surfaces [17]. The symptoms in humans are often vague, but they can also look like those of bronchial pneumonia or even lung neoplasia [18].

Until now, few demonstrations of anthelmintic efficacy against these parasites in hedgehogs were available. Levamisole has been recommended through subcutaneous (SC) injections, as has ivermectin [19]. Nonetheless, using levamisole combined with ivermectin has not resulted in any additional efficacy in comparison to levamisole alone [3]. In another publication, ivermectin administered SC at a high dose was thought to be successful [20], but this was not supported by a more recent study [3].

As for other treatment approaches, previous attempts at treating lungworms in this animal species using moxidectin, either as a spot-on or injectable, even at high dosages, had poor efficacy and were not recommended [3]. Furthermore, oral levamisole for 2 consecutive days has been found to be more effective than a single SC injection of ivermectin; however, an additional dose of levamisole on days 10–12 post-treatment may be indicated, especially when *Crenosoma* spp. or mixed infections are detected and respiratory clinical signs are persistent [1,3]. A recent study concluded that fenbendazole achieved 100% efficacy in treating *Capillaria* spp. in *E. roumanicus*; however, the sample size was very small (five animals) and the dosage of fenbendazole used was very high (100 mg/kg). It is important to mention that hedgehogs that present with clinical signs of respiratory disease should also be treated with antimicrobials, bronchodilators, mucolytics, and supportive fluid therapy, in addition to anthelminthics [3].

The current study presented, for the first time, a successful, safe, and easy-to-administer treatment protocol for infection by *Crenosoma* spp. and *Capillaria* spp. in white-breasted hedgehogs. It demonstrated the efficacy and safety of a single oral administration of the combination of esafoxolaner, eprinomectin, and praziquantel against lungworm infections in hedgehogs. All the medicated animals were lungworm-free on days 10 and 14 post-treatment. The oral route was chosen because the absorption from the hedgehogs’ thickened skin was questioned.

This combination of active compounds could be effective against most hedgehog parasites, not just lungworms, as it is actually an endectoparasiticide. This renders NexGard^®^ Combo a suitable therapeutic approach against multiple parasites, which is often the case in hedgehogs. More specifically, esafoxolaner is an insecticide and acaricide that works by blocking chloride channel activity [21]. Eprinomectin belongs to the class of avermectins and acts by binding to chloride ion channels on the nerves and muscular cells of nematodes while it also kills their larvae, and praziquantel is a pyrazino-isoquinoline derivative anthelminthic with well-established trematocidal and cestocidal action [22].

Another point of interest is that the results of the current survey could also suggest a treatment option for the parasites of African pygmy hedgehogs (*Atelerix albiventris*), which have lately gained increasing popularity as pets [23], and this is something that warrants further investigation.

## 5. Conclusions

The current study demonstrates the high prevalence of lungworm parasitism in northern white-breasted hedgehogs in Greece. Verminous pneumonias by the lungworms *C. striatum* and *C. aerophila* is one of the most important threats that hedgehogs face, and the reduction in lungworm burden plays a significant role in the conservation of this animal species. Towards this end, the current study showed that a single oral administration of esafoxolaner, eprinomectin, and praziquantel successfully treats these infections.

## Figures and Tables

**Table 1:** Differences in body weight measurements (grams) of the 14 treated hedgehogs on days 0 and 15 of the study (*p* value < 0.0001).

Hedgehog	BW Day 0 (g)	BW Day 15 (g)	*p* Value
1	637.0	747.0	
2	717.0	896.0	
3	716.0	834.0	
4	797.0	1075.0	
5	644.0	871.0	
6	780.0	955.0	
7	700.0	854.0	
8	787.0	1035.0	
9	689.0	825.0	
10	710.0	847.0	
11	729.0	871.0	
12	628.0	771.0	
13	736.0	946.0	
14	631.0	855.0	
Mean	707.0	884.0	*p* < 0.0001

## Data Availability

Data are included in the manuscript.
